# KCTD5 and Ubiquitin Proteasome Signaling Are Required for *Helicobacter pylori* Adherence

**DOI:** 10.3389/fcimb.2017.00450

**Published:** 2017-10-24

**Authors:** Alhejandra Álvarez, Felipe Uribe, Jimena Canales, Cristóbal Romero, Andrea Soza, María A. Peña, Marcelo Antonelli, Oscar Almarza, Oscar Cerda, Héctor Toledo

**Affiliations:** ^1^Molecular and Cellular Biology Program, Faculty of Medicine, Instituto de Ciencias Biomédicas (ICBM), Universidad de Chile, Santiago, Chile; ^2^Department of Biological and Chemical Sciences, Faculty of Science, Universidad San Sebastián, Santiago, Chile; ^3^Millennium Nucleus of Ion Channels-Associated Diseases (MiNICAD), Santiago, Chile

**Keywords:** *Helicobacter pylori*, KCTD5, Cullin-3, ubiquitin proteasome system, VacA, CagA

## Abstract

In order to establish infection, bacterial pathogens modulate host cellular processes by using virulence factors, which are delivered from the bacteria to the host cell leading to cellular reprogramming. In this context, several pathogens regulate the ubiquitin proteasome system in order to regulate the cellular effectors required for their successful colonization and persistance. In this study, we investigated how *Helicobacter pylori* affect the ubiquitination of the host proteins to achieve the adherence to the cells, using AGS gastric epithelial cells cultured with *H. pylori* strains, *H. pylori* 26695 and two isogenic mutants *H. pylori cag::cat* and *vacA::apha3*, to characterize the ability of *H. pylori* to reprogram the ubiquitin proteasome systems. The infection assays suggest that the ubiquitination of the total proteins does not change when cells were co-culture with *H. pylori*. We also found that the proteasome activity is necessary for *H. pylori* adhesion to AGS cells and the adherence increases when the level of KCTD5, an adaptor of Cullin-3, decrease. Moreover, we found that KCTD5 is ubiquitinated and degraded by the proteasome system and that CagA and VacA played no role on reducing KCTD5 levels. Furthermore, *H. pylori* impaired KCTD5 ubiquitination and did not increase global proteasome function. These results suggest that *H. pylori* affect the ubiquitin-proteasome system (UPS) to facilitate the adhesion of this microorganism to establish stable colonization in the gastric epithelium and improve our understanding of how *H. pylori* hijack host systems to establish the adherence.

## Introduction

The establishment and progression of infectious diseases depend on interactions between the pathogens and host cells. Therefore, pathogens express specialized factors to overcome the host defense mechanisms. Pathogens modify signaling pathways in host cells to promote their own survival, replication, and escape from host immune responses (Ribet et al., 2010). Diverse post-translational modifications such as phosphorylation, sumoylation and ubiquitination can be induced for this purpose (Ribet and Cossart, 2010). Ubiquitination is a versatile, reversible and dynamic post-translational modification (Chen and Sun, 2009), performed by the hierarchical action of the E1 activating enzyme, E2 conjugating enzyme and E3 ubiquitin ligase (Komander and Rape, 2012). The E3 ubiquitin ligase confers substrate specificity and dictates the nature of the ubiquitin linkage(s), which will determine the substrate fate (Metzger et al., 2014). The reaction results in ubiquitin (Ub) transfer to the amino group of lysine residues of the target protein or to ubiquitin itself in the case of poly-ubiquitination (Hershko and Ciechanover, 1998). Protein ubiquitination pathways are implicated in several cellular functions such as protein degradation, anterograde trafficking, endocytosis, signal transduction, DNA repair and transcriptional regulation (Golubnitschaja, 2007; Konstantinova et al., 2008). Due of the importance of these processes, it is not surprising that a wide variety of human pathogens such as *Salmonella enterica, Listeria monocytogenes, Yersinia enterocolitica, Mycobacterium tuberculosis*, and *Legionella pneumophila*, manipulate the host ubiquitination mechanisms to promote their colonization (Rytkönen and Holden, 2007; Popa et al., 2016). For instance, *Salmonella* type III effectors SopA and SptP modulate members of the ubiquitin-proteasome system (UPS) to affect the host cell cytoskeleton during invasion (McGhie et al., 2009). Conversely, *L. pneumophila* encodes F-box proteins, proteins that may form complexes with the Cullin-RING ubiquitin ligases (CRL) in infected cells (Price and Kwaik, 2010). An example of such effectors is AnkB, which allows the production of free amino acids using the UPS of its host (Price et al., 2011). *Escherichia coli* encodes two E3 ligase enzyme analogs: NIeL and NIeG. NIeL modulates the formation of the actin pedestal that allows the adhesion of *E. coli* to the intestinal epithelial cells (Piscatelli et al., 2011) and in the case NleG, the molecular target in host cells remains unknown (Wu et al., 2010). Moreover, effects on the ubiquitination of cellular proteins by *Helicobacter pylori* infection have been previously reported (Lamb et al., 2009).

*H. pylori* is a microaerophilic, Gram-negative, spiral-shaped, flagellated and neutrophilic microorganism that infect 50% of the worldwide population (Tomb et al., 1997). Moreover, about 10–15% of the infected persons develop peptic ulcer disease, gastric adenocarcinoma, and mucosa-associated lymphoid tissue (MALT) lymphoma (Mitchell and Katelaris, 2016). Thus, the interaction between the bacteria and gastric epithelium and the colonization mechanisms are particularly relevant. Here, the manipulation of host signaling cascades by *H. pylori* appears to be crucial for chronic infection and the onset of gastric disease progression.

Recently, it has been demonstrated that the ubiquitin-proteasome pathway regulates survivin upon *H. pylori* infection (Zhao et al., 2000; Mirza et al., 2002; Valenzuela et al., 2013). Additionally, proteasome-dependent degradation induced by *H. pylori* has been described for p53 and p27, promoting a higher risk to gastric carcinogenesis (Eguchi et al., 2004; Wei et al., 2010). As was reported for p53 (Wei et al., 2010), and TAK1 (Lamb et al., 2009, 2013), this degradation mechanism is correlated with an increase of ubiquitination of these proteins. However, no further targets or role for ubiquitination and proteasome-dependent degradation during *H. pylori* colonization has been identified. Interestingly, microarrays analyses performed with gastric biopsies from *H. pylori*-infected patients have shown an increase in ubiquitin and Cullin-3 (CUL3) E3 ligase expression (Galamb et al., 2008). CUL3 is the most prevalent E3 ligase of the CRL family (Genschik et al., 2013), which may be responsible for 20% of proteasome-dependent protein turnover (Soucy et al., 2009). CRLs are composed of several modular subunits that include an adaptor that recognizes the substrate, a large connector denoted as Cullin and a RING protein that binds the E2 enzyme (Balasco et al., 2014). In the case of the CUL3 CRL, the substrate-specific adaptors are proteins containing a BTB (Bric-a-brac, Tramtrack and Broad Complex) domain, a common protein that mediates protein-protein interactions (Furukawa et al., 2003; Pintard et al., 2003; Xu et al., 2003). In humans, there are around 200 proteins with BTB domain, however not all proteins containing a BTB domain interacts with CUL3 (Pintard et al., 2004; Smaldone et al., 2015). Within the proteins with BTB domain is the family of potassium channel tetramerization domain (KCTD), the name of the protein family is given by the similarity of tetramerization domain in some voltage-gated potassium channels and the conserved N-terminal region of KCTD proteins (Liu et al., 2013). These proteins regulate diverse functions such as transcriptional repression, cytoskeletal remodeling and ion channels gating (Liu et al., 2013). Several members of KCTD family have been shown to interact with CUL3 CRL (Canettieri et al., 2010; Lange et al., 2012; Kim et al., 2017). One of these proteins, KCTD5, interacts with CUL3 and ubiquitinated proteins, suggesting that this member of KCTD family might play a role as a substrate-specific adaptor for CUL3 E3 ligase (Bayón et al., 2008). Nevertheless, the mechanisms and cellular processes in which KCTD5 participates remain unclear. Moreover, possible targets of ubiquitination and proteasomal degradation induced by the CUL3/KCTD5 complex are still unknown. Herein, we raised the question, whether the *H. pylori* infection impact the ubiquitin proteasome system and the participation of KCTD5 CUL3 adaptor during the adherence to epithelial cells.

## Materials and methods

### Cell lines, culture conditions, and plasmids

AGS human gastric epithelial cells (CRL-1739; American Type Culture Collection, Manassas, VA) were grown at 37°C, 5.0% CO_2_ and 80% relative humidity (RH) in RPMI 1640 media (Thermo Fisher Scientific, Suwanee, GA, USA) supplemented with 10% v/v fetal bovine serum (Invitrogen, Carlsbad, CA, USA). Cells were transiently transfected with plasmids using Lipofectamine LTX (Invitrogen) according the manufacturer instructions. HEK293T cells (CRL-1573; American Type Culture Collection, Manassas, VA) were cultured in DMEM medium (Thermo Fisher Scientific) supplemented with 5% v/v fetal bovine serum at 37°C, 5% CO_2_ and 80% RH and, transfected with Lipofectamine 2000 according to the manufacturer's instructions.

Plasmids encoding Hemagglutinin-tagged (HA-Ubi) and Histidine-tagged Ubiquitin (His-Ubi) were generous gifts by Drs. Edward Yeh and Astar Winoto (*via* Addgene plasmids 18712 and 31815, respectively). EGFP-fused KCTD5 encoding plasmid (pEGFP-C1-KCTD5) was generated by cloning the human KCTD5 cDNA from the pMaxKoz-HA-KCTD5 plasmid (Dementieva et al., 2009) (kindly donated by Dr. Steve Goldstein) into the XhoI/BamHI sites of the pEGFP-C1 plasmid (Catalog number: 6084, Clontech Laboratories, Mountain View, CA, USA). KCTD5 shRNA (shRNA^#Scramble^ and shRNA^#KCTD5^) were obtained from Origene Inc. (Origene, Rockville, MD, USA).

### *H. pylori* strains and culture conditions

*H. pylori* strains 26695 (ATCC 700392), a *cagA*-positive and *vacA*-positive strain, and its isogenic mutants *H. pylori cagA::cat* and *vacA::apha3* were grown as described before, on tripticase soy agar (TSA) (Becton-Dickinson, Sparks, MD, USA) plates supplemented with 5% v/v horse serum (Corning Life Sciences, Tewksbury, MA, USA), the culture supplement Vitox (Oxoid Basingstoke, Hampshire, England), and the antibiotic supplement Dent (Oxoid) for 24–48 h at 37°C in a microaerophilic condition (6.5% O_2_; 5.5% CO_2_ and 70%-80% RH) (25). *H. pylori vacA::apha3* was developed in this study.

### Adherence assay of *H. pylori* to AGS cells

One hundred thousand AGS cells were seeded in a 24-well plate. Eight hours co-culture with *H. pylori* (MOI = 300) in the presence and absence of 2.5 μM Lactacystin, an irreversible proteasome inhibitor, were performed as described (Coombs et al., 2011). After infection cells were washed three times with 1X DPBS to remove not adhered bacteria. Then, cells were incubated with 0.1% w/v saponin in 1X DPBS for 10 min at 37°C and 5% CO_2_. The lysate was recovered and serial dilutions were made for counting of colony forming units on TSA plates.

To evaluate the effect of KCTD5 on *H. pylori* adherence to AGS cells the KCTD5 protein was overexpressed by transfecting the AGS cells 48 h prior to the infection with pEGFP-C1-KCTD5 vector. pEGFP-C1 plasmid was used as a control. KCTD5 knockdown was performed by transfecting the cells with the shRNA^#Scramble^ or shRNA^#KCTD5^ 72 h prior the infection.

### Growth of *H. pylori* in the presence of lactacystin

To determine whether the proteasome inhibitor Lactacystin affects the viability of *H. pylori*, the bacterial growth curves were performed in trypticase soybean broth and microaerophilic condition at 37°C with shaking (180 rpm) in presence and absence of 2.5 μM Lactacystin. The culture was started with an inoculum of 0.2 U, OD 560 nm, of *H. pylori* suspension in 1X DPBS previously grown for 48 h. Samples were taken at 0, 6, 9, 24, and 48 h to construct the growth curve.

### *In Vitro* proteasome activity assay

AGS cells were infected for 8 h with *H. pylori* wild type, *cagA*^−^ and *vacA*^−^ strains. Then, 20 μg aliquots of cell extracts were incubated in reaction buffer [50 mM Tris-HCl (pH 7.5), 1 mM dithiothreitol (DDT) and, 0.5 mM EDTA] in triplicate for 60 min at 37°C with the fluorogenic substrate Trypsin-like Z-Leu-Leu-Glu-AMC (Calbiochem, San Diego, CA, USA). The amount of product (free AMC) was measured by Fluorocounter, GloMax®-Multi (Promega, Madison, Wisconsin, USA), with an excitation filter of 365 nm and an emission filter of 460 nm (Mlynarczuk-Bialy et al., 2014).

### Proteasome and lysosome inhibition

Proteasome and lysosome inhibition were performed in the presence of Lactacystin (2.5 μM) and Chloroquine (100 μM), respectively. In both cases, the AGS cells were incubated for 30 min in the presence of the inhibitors. Then a co-culture of the cells with *H. pylori* was done for 8 h, after this time a cell extract was obtained and analyzed by immunoblot.

### KCTD5 half-life analysis

AGS cells were co-cultured for 6 h with *H. pylori*, then 100 μg/mL cycloheximide was added. Cell lysates were obtained at intervals of 30 min for 3.5 h, and KCTD5 levels were determined by immunoblot as described (Cáceres et al., 2015).

### Immunoblot analysis and quantification

Total levels of total ubiquitinated proteins was determined by immunoblot assays as described (Cáceres et al., 2015) using anti-ubiquitin mouse monoclonal (Catalog number: AUB01, Cytoskeleton, Inc., Acoma ST, Denver, USA), anti-CUL3 rabbit polyclonal (Catalog number: TA326917, Origene) and anti-KCTD5 mouse monoclonal (Catalog number: TA501035, Origene) antibodies. Anti-Tubulin mouse monoclonal (Catalog number: T5168, Sigma-Aldrich, St Louis, Missouri, USA) and anti-CagA mouse monoclonal antibodies (Catalog number: sc-28368, Santa Cruz Biotechnology, Dallas, Texas, USA) were used as loading and *H. pylori*-infection controls, respectively. Immunoblots were visualized by Pierce ECL Western Blotting Substrate (Thermo Fisher Scientific). Images were acquired with a MiniHD9 (UVITEC, Cambridge) chemoluminiscence photodocumentation system and quantified using NIH/ImageJ software.

### Ni^2+^-NTA column affinity chromatography

HEK293T and AGS cells were transfected with the EGFP, EGFP-KCTD5 and His-Ubi plasmids. HEK293T cells were treated with 1.5 μM Lactacystin after 36 h post-transfection, and AGS cells with 2.5 μM Lactacystin 30 min before *H. pylori* infection. After 48 h, cells were solubilized in lysis buffer (0.5% v/v NP-40, 300 mM NaCl, 50 mM Tris-HCl (pH 8.0), 5 mM NaF, 40 mM Imidazole, 1 mM Phenylmethylsulfonyl fluoride (PMSF) and 1 mM Protease Inhibitor Cocktail) and incubated by 30 min at 4°C, then the lysate were centrifuged at 11,000 × g for 10 min at 4°C. One hundred microliters from protein extracts were separated as input and the remanent was incubated with Ni^2+^-NTA affinity columns for 2 h at 4°C with gentle shaking. The columns were then washed 8 times with wash buffer (0.5% v/v NP-40, 300 mM NaCl, 50 mM Tris-HCl (pH 8.0), 5 mM NaF, 40 mM Imidazole). Subsequently, the proteins were eluted with 5x Reducing Sample Buffer and finally resolved by SDS-PAGE electrophoresis and analyzed by immunoblot.

### Statistical analysis

Statistical analyzes were performed with the GraphPad Prism program, version 6.01 for Windows® (GraphPad Software, Inc. La Jolla, CA 92037, USA). The statistical test, *t*-Student, was used with a significance of *p* < 0.05.

## Results

### *H. pylori* infection decreases proteasome activity

**Figure 1:**
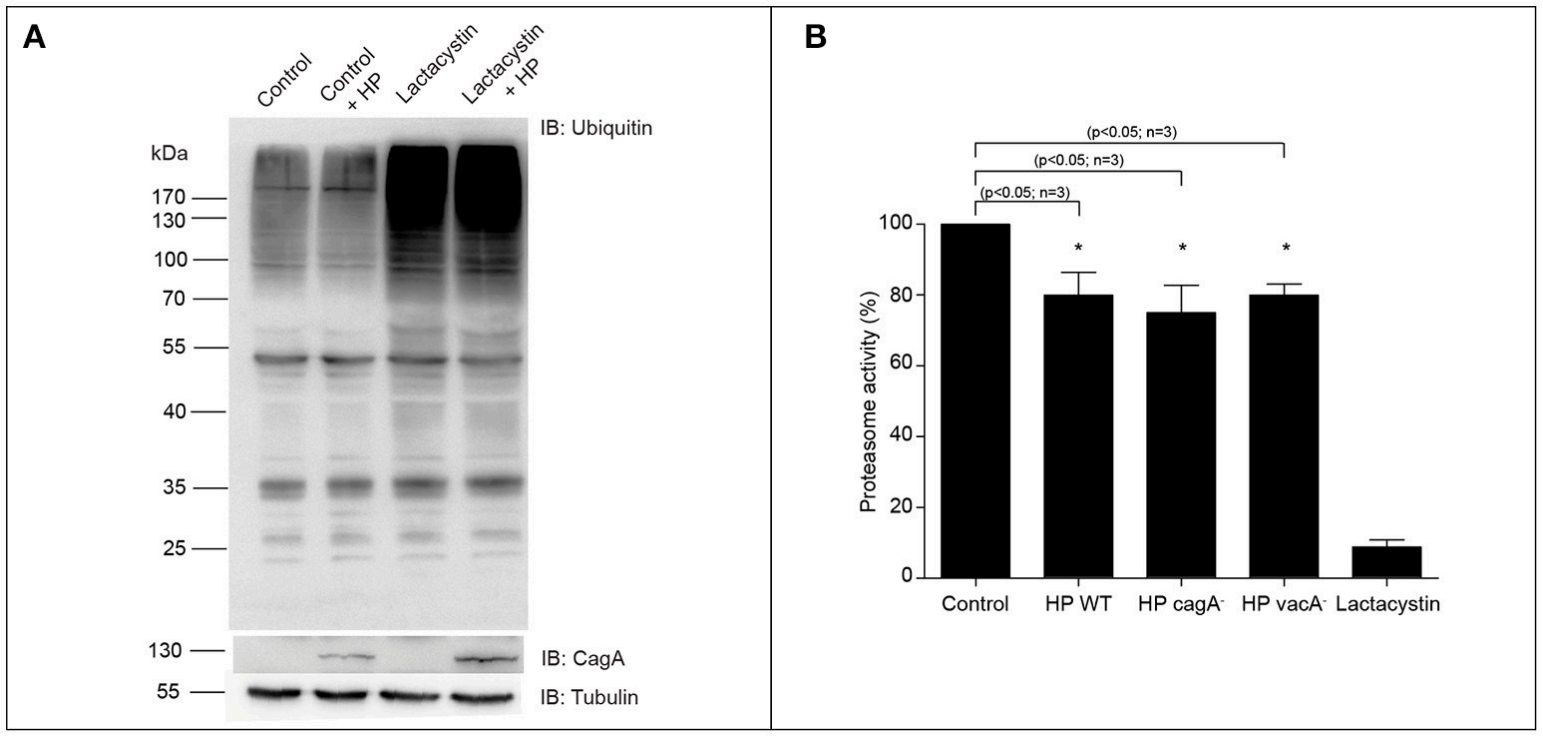
Ubiquitination of total proteins and proteasome activity in AGS cells co-cultured with *H. pylori*. **(A)** Representative immunoblot of total ubiquitinated protein from AGS cells infected with *H. pylori* (MOI = 300) for 8 h, in presence or absence of 2.5 μM Lactacystin. Tubulin is shown as loading control and CagA as infection control. **(B)** Quantification of proteasome activity in AGS cells infected with *H. pylori* wild type and its isogenic mutants *cagA, vacA* and, in presence of 2.5 μM Lactacystin for 8 h. Asterisk represents significant difference (*p* < 0.05). Statistical analysis was performed using a Student's *t*-test. Error bars show standard deviation.

In order to determine whether *H. pylori* colonization induces differential ubiquitination, we transfected AGS cells with a histidine-tagged ubiquitin plasmid and that cells were infected with wild type *H. pylori* for 8 h (MOI = 100). Then cells were lysed and the total protein extract analyzed by immunoblot. We observed that *H. pylori* infection causes an increase in the ubiquitination of the host proteins (Figure S1). Previous reports showed an increase in the ubiquitination of the proteins upon *H. pylori* infection in the AGS cells transfected with HA epitope-tagged ubiquitin (HA-Ubi) incubated with the proteasome inhibitor MG-132 (Yu et al., 2013). In order to determine whether *H. pylori* colonization induces overall ubiquitination in AGS cells, we analyzed the endogenous ubiquitination in these cells. To do that, we infected the AGS cells with *H. pylori* (MOI = 300) for 8 h. Then, cells were lysed and the total protein extracts were analyzed by immunoblot. We observed that *H. pylori* infection does not modify the levels of total ubiquitinated proteins (Figure 1A). However, when we analyzed the activity of the proteasome during the infection of *H. pylori*, an approximately 20% decrease in the proteasome activity was observed (Figure 1B). This effect was also observed when the infection was done with the *cagA*^−^ and *vacA*^−^ *H. pylori* mutant strains. Then, the proteasome decreased activity is independent of those virulence factors (Figure 1B). These data suggest that *H. pylori* affects the ubiquitination signaling during the infection in a differential manner.

### Proteasomal inhibition decreases *H. pylori* adherence to gastric cells

**Figure 2:**
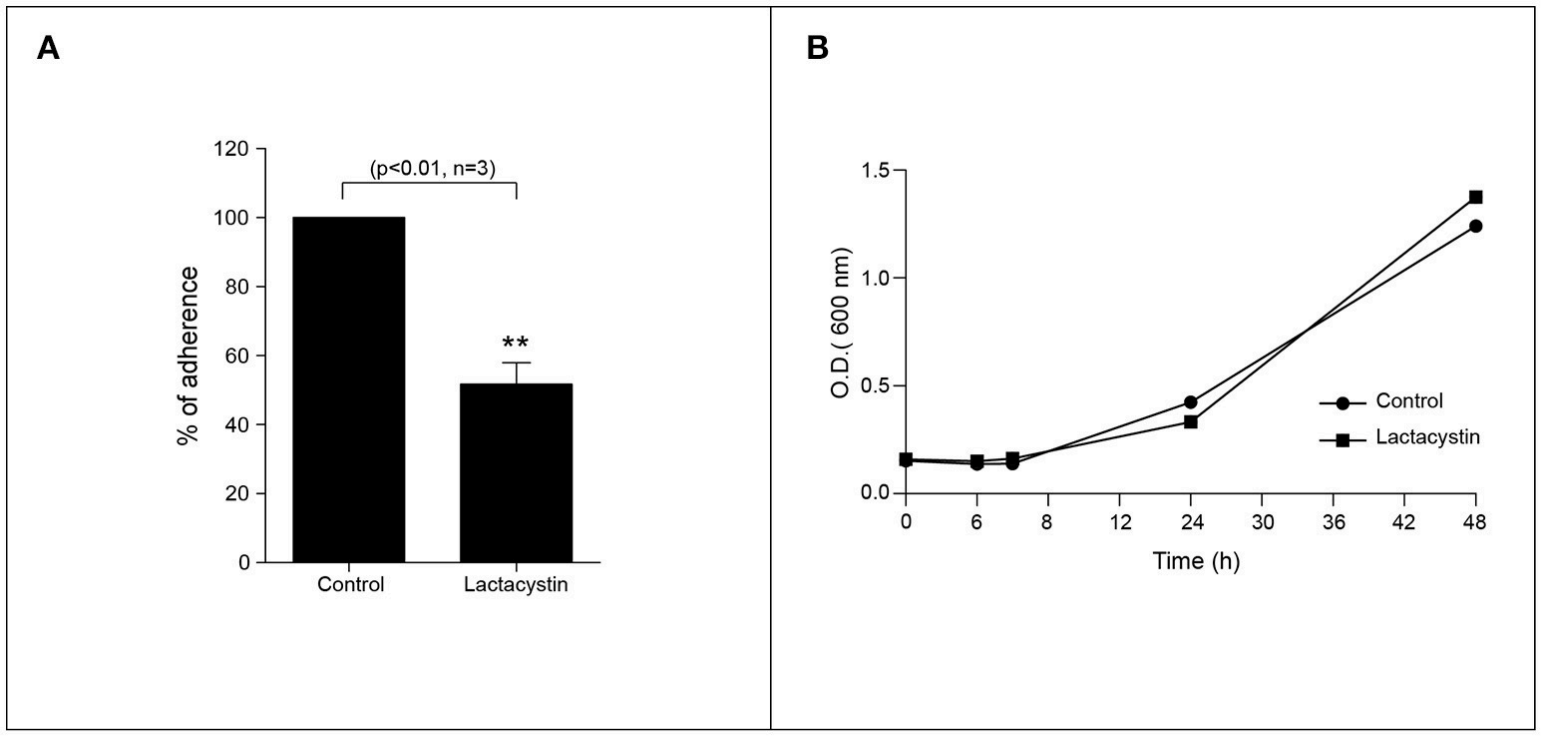
Proteasome inhibition reduces the *H. pylori* adhesion to AGS cells. **(A)** Quantification of the *H. pylori* adhered to AGS cell in presence or absence of the 2.5 μM Lactacystin. Asterisk represents significant difference (*p* < 0.01). Statistical analysis was performed using a Student's *t*-test. Error bars show standard deviation. **(B)** Growth curve of *H. pylori* in presence (square) or absence (circle) of 2.5 μM lactacystin.

UPS is one of the most remarkable protein degradation pathways related to ubiquitinated proteins (Lee and Goldberg, 1998). However, the role of proteasome activity for *H. pylori* colonization has not been yet demonstrated. We then assessed to explore the effect of pharmacological inhibition of the proteasome in the *H. pylori* adhesion. To do that, we infected AGS cells with *H. pylori* (MOI = 300) for 8 h in presence or absence of 2.5 μM Lactacystin. Interestingly, we observed that proteasome inhibition reduced in a ~50% the *H. pylori* adhesion to AGS cells (Figure 2A). In order to discard a possible bacteriostatic or bactericidal effect of the Lactacystin treatment, we incubated *H. pylori* cultures with 2.5 μM Lactacystin, and we evaluated its effect during bacterial growth. As shown (Figure 2B) no significant differences in Lactacystin treated and control growth curves were observed. Moreover, bacteria morphology does not change upon Lactacystin treatment (Figure S2A). These data suggest that Lactacystin treatment did not affect the growth and viability of *H. pylori*. Moreover, the protective effect of Lactacystin was not related to a bactericidal/bacteriostatic effect, suggesting that the proteasome machinery of the host cells is required for the proper infection of *H. pylori*. Similar results were observed with MG-132, another pharmacological proteasome inhibitor (Figure S2B).

### *H. pylori* infection does not induce changes in the levels of CUL3

**Figure 3:**
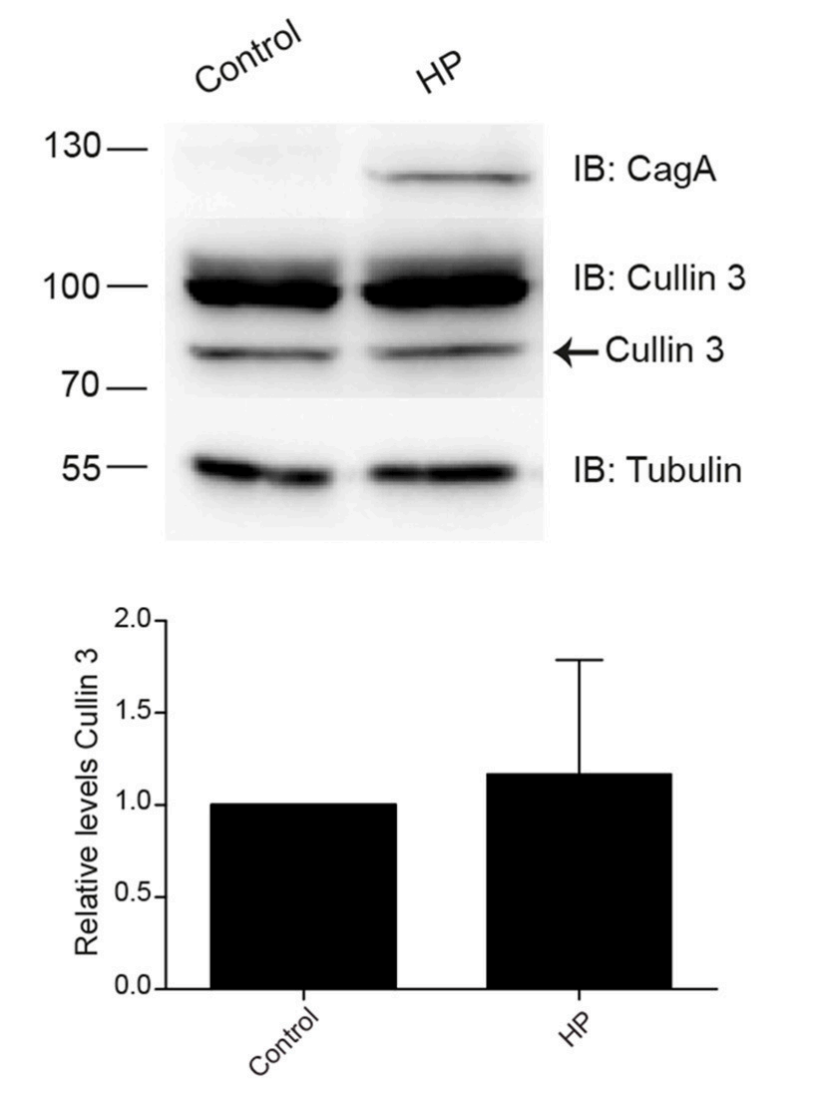
Levels of CUL3 in AGS cells infected with *H. pylori*. Representative immunoblot and quantification of the CUL3 levels determination in AGC cells infected with *H. pylori*. Statistical analysis was performed using a Student's *t*-test (*n* = 5; *p* < 0.5802). Error bars show standard deviation.

Microarrays analyses performed with gastric biopsies from *H. pylori*-infected patients had shown an increase in ubiquitin and CUL3 E3 ligase expression (Galamb et al., 2008). Since these changes in protein expression might be involved in *H. pylori* adhesion, we evaluate the CUL3 expression upon *H. pylori* infection. However, when we analyzed the levels of CUL3 in AGS *H. pylori*-infected cells we did not observe changes in the protein level (Figure 3).

### KCTD5 is degraded during *H. pylori* infection and its degradation enhanced the adherence of the bacteria

**Figure 4:**
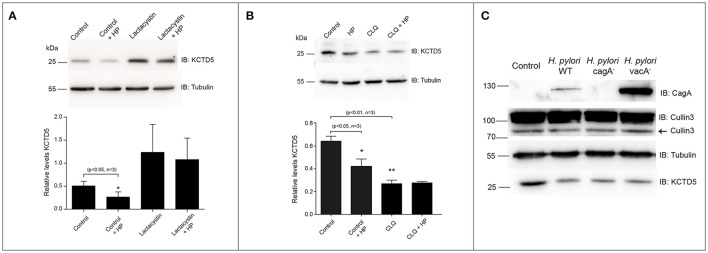
KCTD5 proteins in AGS cells infected with *H. pylori*. **(A)** Representative immunoblot and quantification of KCTD5 levels in AGS cells infected with *H. pylori* wild type in presence or absence of 2.5 μM Lactacystin. **(B)** Determination of KCTD5 levels during *H. pylori* infection in presence of 100 μM Chloroquine for 8 h**. (C)** Representative immunoblot showing levels of CUL3 and KCTD5 in AGS cells infected with *H. pylori* wild type or its isogenic mutants *cagA* and *vacA*. It also shows the presence of CagA in extracts of AGS cells infected with the bacterium as an infection control. Asterisk represents significant difference (*p* < 0.05) vs. untreated control. Statistical analysis was performed using a Student's *t*-test.

**Figure 5:**
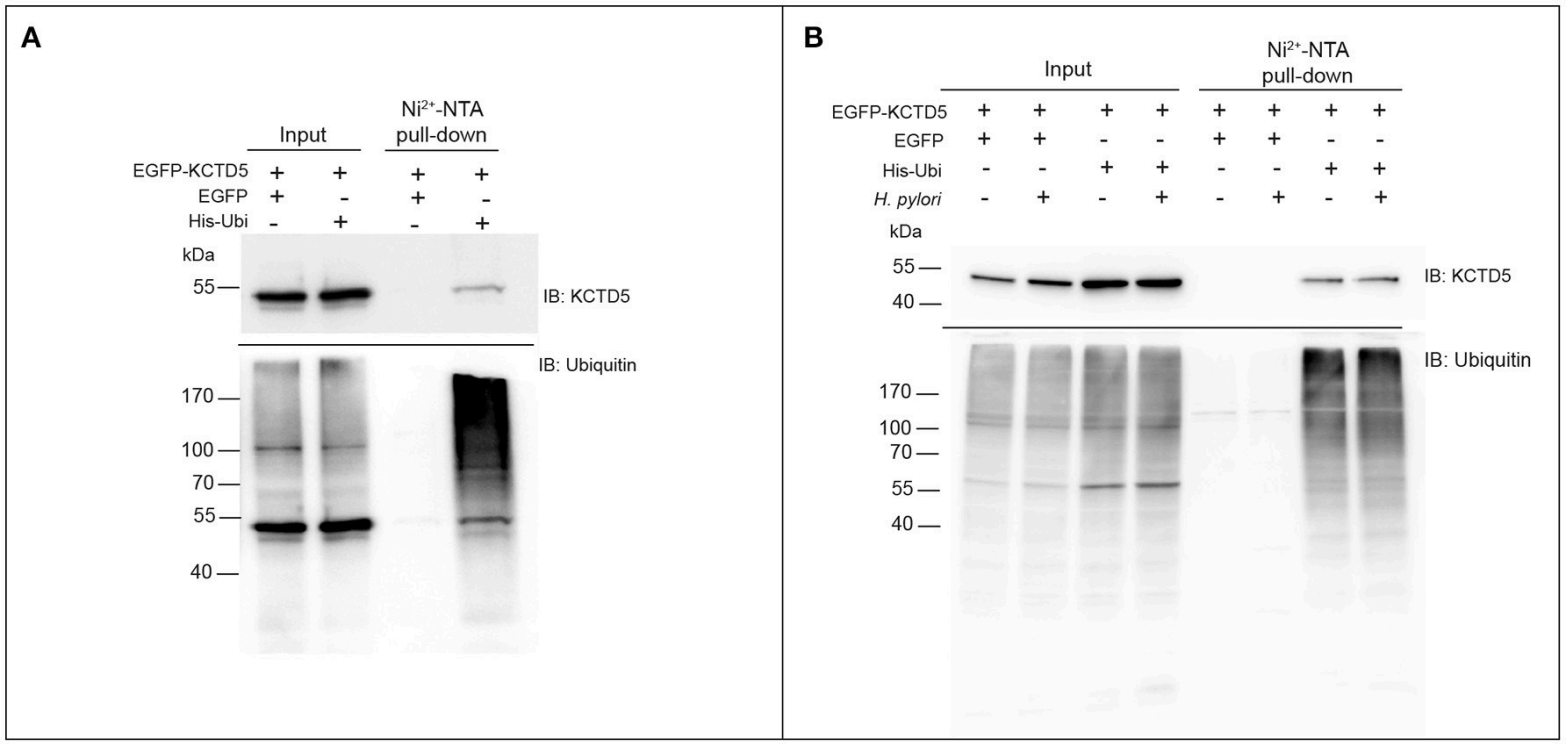
Retention of KCTD5 in the Ni^2+^-NTA column during *H. pylori* infection. **(A)** HEK293T and **(B)** AGS cells were transfected with plasmids encoding EGFP, EGFP-KCTD5 and His-Ubi, lysates from these cells were processed for pull-down with Ni^2+^-NTA column, and the presence of KCTD5 was determined by immunoblot with antibody to KCTD5 (upper panel) and ubiquitinated proteins were shown as retention control in the column (lower panel). AGS transfected cells were infected by 8 h with *H. pylori* (MOI = 300).

We next achieve to determine whether *H. pylori* infection alters the levels of KCTD5, a CUL3 adapter. To do that, *H. pylori*-infected AGS cells lysates were analyzed by immunoblot. In these experiments, we observed decreased levels of KCTD5 protein, due to proteasome-dependent degradation (Figure 4A). Moreover, treatments with a lysosomal degradation inhibitor (i.e., chloroquine) decrease the KCTD5 levels in a *H. pylori*-independent manner (Figure 4B). These results suggest the role of proteasome in KCTD5 degradation. Also, KCTD5 degradation was not affected by CagA or VacA (Figure 4C). We next verified whether KCTD5 is ubiquitinated (Figure 5A). To do that, we transfected HEK293T and AGS cells with a Histidine-tagged version of ubiquitin (His-Ubi) plasmid, or with pEGFP-C1 as control. Both transfections were co-transfected with pEGFP-C1-KCTD5 in the presence of a proteasome inhibitor. In these experiments, KCTD5 is observed on Ni^2+^ column His-Ubi containing eluates, showing the ubiquitination of KCTD5 (Figure 5A, upper). Moreover, we observed that apparently *H. pylori* infection does not increases the ubiquitination of KCTD5, as was observed in the Ni^2+^ column-retained fractions (Figure 5B). Therefore, KCTD5 is degraded *via* proteasome upon *H. pylori* infection, with no overall increase on its ubiquitination.

**Figure 6:**
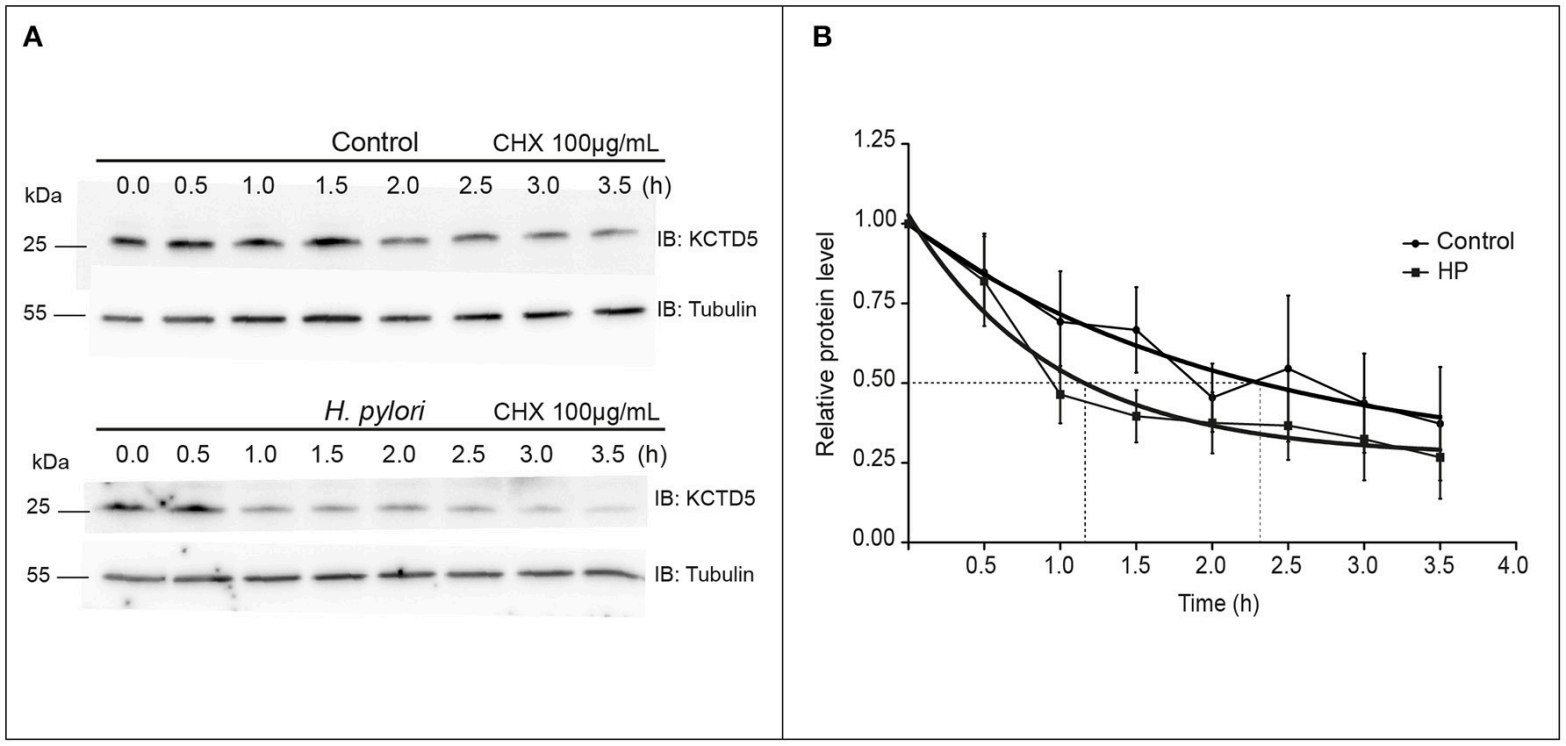
Determination of the KCTD5 half-life. **(A)** Determination of the KCTD5 half-life was performed by immunoblot measuring the levels of KCTD5 in *H. pylori* infected AGS cells over a time interval of 3.5 h at intervals of 30 min. **(B)** The curves represent the average of three independent cultures. Control (circle); AGS cells infected with *H. pylori* (squared). Error bars show standard error of the mean (SEM).

**Figure 7:**
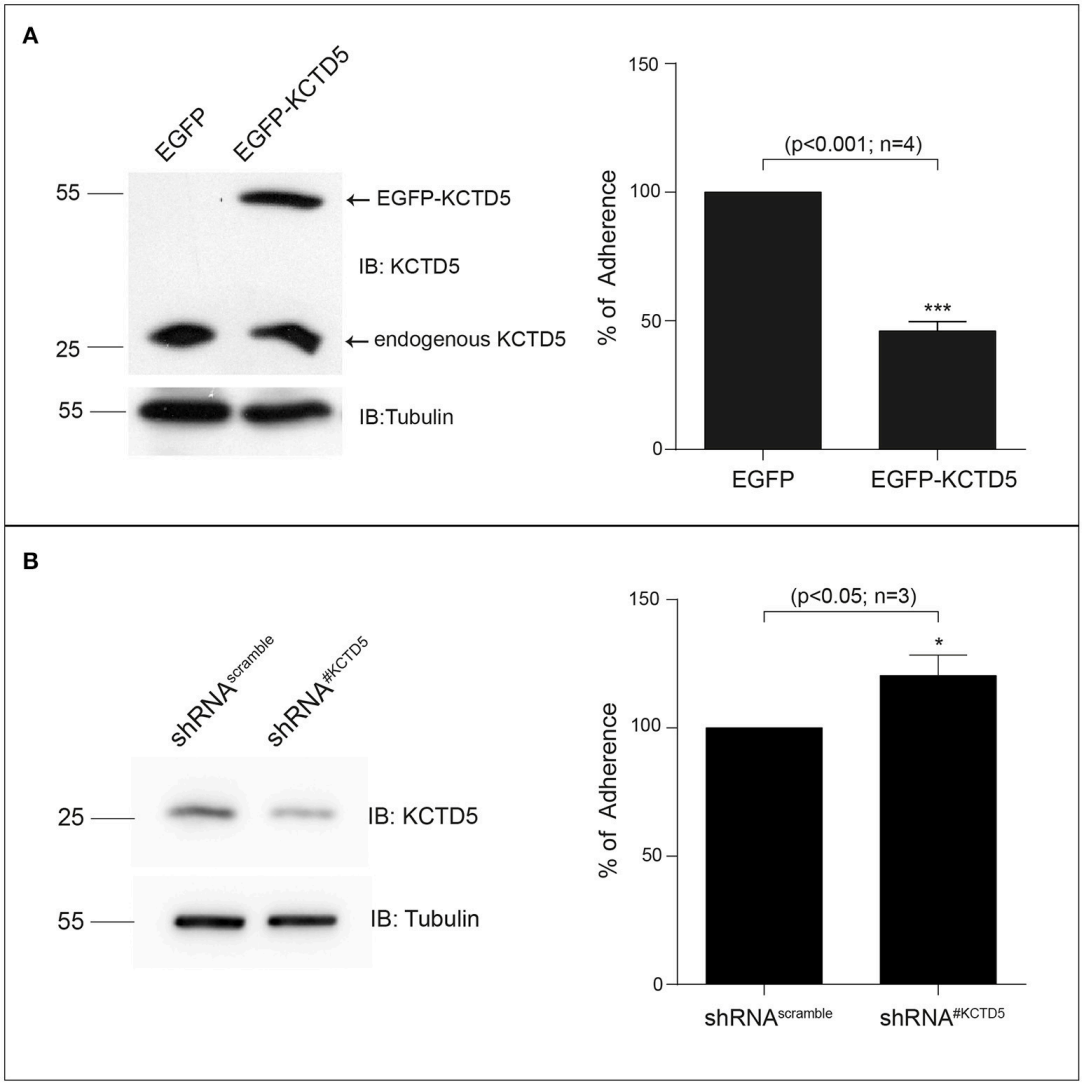
KCTD5 expression modulates the *H. pylori* adhesion to AGS cells. **(A)** Representative immunoblot of the overexpression of pEGFP-C1-KCTD5 in AGS cells (left). Four independent adhesion experiments were performed by incubating AGS cells, with *H. pylori* (right) **(B)** Representative immunoblot of the KCTD5 knockdown in AGS cells (left). Three independent adhesion experiments were performed by incubating AGS cells, transfected with shRNA^#Scramble^ or shRNA^#KCTD5^, with *H. pylori*. Statistical analysis was performed using a Student's *t*-test. Error bars show standard deviation.

We then analyzed the kinetics of degradation of KCTD5 during the *H. pylori* infection. Here, we observed that the half-life of KCTD5 was 1.2 h compared to 2.4 h with the non-infected culture (Figure 6) suggesting that *H. pylori* adherence induces KCTD5 degradation. To determine whether KCTD5 contributes to *H. pylori* adhesion, we overexpressed KCTD5 protein in AGS cells for 48 h. Then, cells were infected with *H. pylori* for 8 h. In these experiments, we found a ~50% decrease in the adherence of *H. pylori* (Figure 7A). To determine whether KCTD5 degradation was involved in the adherence of *H. pylori* to AGS cells we used a loss-of-function approach. Endogenous KCTD5 expression in the AGS cells was silenced with the shRNA^#KCTD5^ and we observed a ~20% increase in the *H. pylori* adherence (Figure 7B). Together, these data confirm that KCTD5 degradation mediates the *H. pylori* adherence to AGS cells. Lower levels of mRNA and KCTD5 protein increased the adherence of *H. pylori* to AGS cells.

## Discussion

*H. pylori* adherence to the gastric epithelium cells is one of the most relevant stages for a successful chronic colonization (Oleastro and Ménard, 2013). Recent advances have allowed a better understanding of *H. pylori* pathogenicity-related factors and their interactions with gastric epithelial cell components (Pachathundikandi et al., 2013; Alzahrani et al., 2014). Bacteria adhesion triggers a plethora of cellular changes that involve several signal transduction cascades, favoring the persistance of the microorganism in the gastric mucosa (Johnson et al., 2012), activation of NF-κB and secretion of interleukin-8 (IL-8) (Rieder et al., 1997). During infection, *H. pylori* is in close contact with the epithelial cell membrane, with the formation of adhesion pedestals (Hessey et al., 1990). Moreover, *H. pylori* binds to gastric epithelial cells inducing changes in the cytoskeleton (Smoot et al., 1993). To do that, bacteria that successfully colonize the mucosa usually express specialized factors to adhere to the epithelial cells. *H. pylori* express molecules such as BabA or SabA with affinity for glycoconjugates on the membrane of the epithelial cells that participate in the adherence mechanisms (Mahdavi et al., 2002). Also, cell-associated bacteria alter gastric epithelial cell behavior through CagA, VacA, and CagL. CagL, a gene member of the *cag* pathogenicity island, was suggested to function as a tip adhesin that binds to α5β1 integrin through a RGD motif (Salama et al., 2013). Conversely, other *H. pylori*-host interactions are established with other cell surface receptors, such as αvβ5 integrin (Kwok et al., 2007; Pachathundikandi et al., 2013). In this study, we aimed to examine the adhesion mechanism of *H. pylori* to AGS cells. We found that *H. pylori* infection induces changes in the UPS. Our results show that *H. pylori* infection induces a decrease in proteasome activity. These results are consistent with the data reported by Eguchi et al. (2003), who also observed a decrease of the proteasome activity on AGS cells infected with *H. pylori* (Eguchi et al., 2003). In addition, we observed that the virulence factors VacA and CagA do not have a role on the induction of this effect. Then, the virulence factor involved in the manipulation of the UPS system remains to be determined.

We also determined the effect of proteasome inhibition on *H. pylori* adherence. It was consistently found that pharmacological inhibition of the proteasome decreases the adherence of *H. pylori* on epithelial cells. That result suggests that proteolysis of some proteins might be required for cytoskeleton reorganization and/or for the pedestal adherence organization building in order to allow an effective adhesion. With this information, we can infer that the protein degradation is important to carry out the process of bacterial infection. Data from other bacteria such as *Streptococcus pneumoniae* support this idea, since the inhibition of the ubiquitin proteasome system increases the survival of this intracellular bacterium in its cellular host (Iovino et al., 2014).

In order to clarify alterations in the UPS during *H. pylori* infection, we measured the levels of the CUL3 ligase protein, which gene expression has been reported to be increased during infection (Galamb et al., 2008). However, we did not observe significant differences in CUL3 protein levels due to *H. pylori* infection under our experimental conditions (Figure 3). On the other hand, we analyzed the levels of one of the CUL3 adapters, KCTD5 (Bayón et al., 2008), a protein whose function is to recognize the substrate to be modified. Interestingly, we found that KCTD5 levels decreased during infection in a CagA and VacA-independent manner (Figure 4C). Moreover, we observed an increase on KCTD5 levels by inhibiting the proteasome activity (Figure 4A). Therefore, KCTD5 is degraded through proteasome. Also, we verified the KCTD5 ubiquitination, as it was observed from the retained protein in the Ni^2+^-NTA column in HEK293T cells (Figure 5A) and AGS *H. pylori*-infected cells (Figure 5B). Since we do not observe an increase in the ubiquitination of KCTD5 due to the infection with *H. pylori*, we cannot ruled out another ubiquitin-independent mechanism of proteolysis involved on the degradation of KCTD5 (Tofaris et al., 2001; Asher et al., 2005; Ben-Nissan and Sharon, 2014). Thus, the role of ubiquitination in KCTD5 activity remains to be clarified.

KCTD5 is the protein that recognizes the substrate in the CUL3/E3 ligase complex, changes in the CUL3 levels might not be critical for the *H. pylori*-induced effects. In addition, KCTD5 could interact with other scaffolding proteins to form E3 ligase complex during infection. Thus, KCTD5 activity could be a key target during *H. pylori* adhesion. Conversely, chloroquine treatment causes a decrease in KCTD5 levels, but this effect was independent of *H. pylori* infection. This result supports that KCTD5 is degraded by the proteasome and that some protein that could mediate its degradation remains present under chloroquine treatment. In this context, we found that KCTD5 was degraded by the proteasome and its degradation favors the adherence of *H. pylori*. These data suggest that KCTD5, and/or other proteins proteolysis is necessary to facilitate the adherence. This observation is confirmed by the experiments performed with the KCTD5 silencing that showed an increase in the adherence of this microorganism to the AGS cells (Figure 7B) and on the other hand with the overexpression of KCTD5 that induces the opposite effect (Figure 7A).

Interestingly, *H. pylori* infection only affected KCTD5 levels, but not CUL3 levels. KCTD5 has been reported to interact with “the Golgi matrix proteins” GRASP55 (Dementieva et al., 2009). On the other hand, it was observed that the lack of GRASPs produces disruption of the Golgi structure, resulting in accelerated protein trafficking and defective glycosylation (Zhang and Wang, 2015). In addition, in a *Drosophila* model, a GRASP-like protein mediated the trafficking of integrins (Wang et al., 2015). In view of these observations, we hypothesized that a decrease in the levels of KCTD5 could alter the activity of GRASP55, and thus regulate the trafficking of integrins, which could promote the adhesion of *H. pylori*.

**Figure 8:**
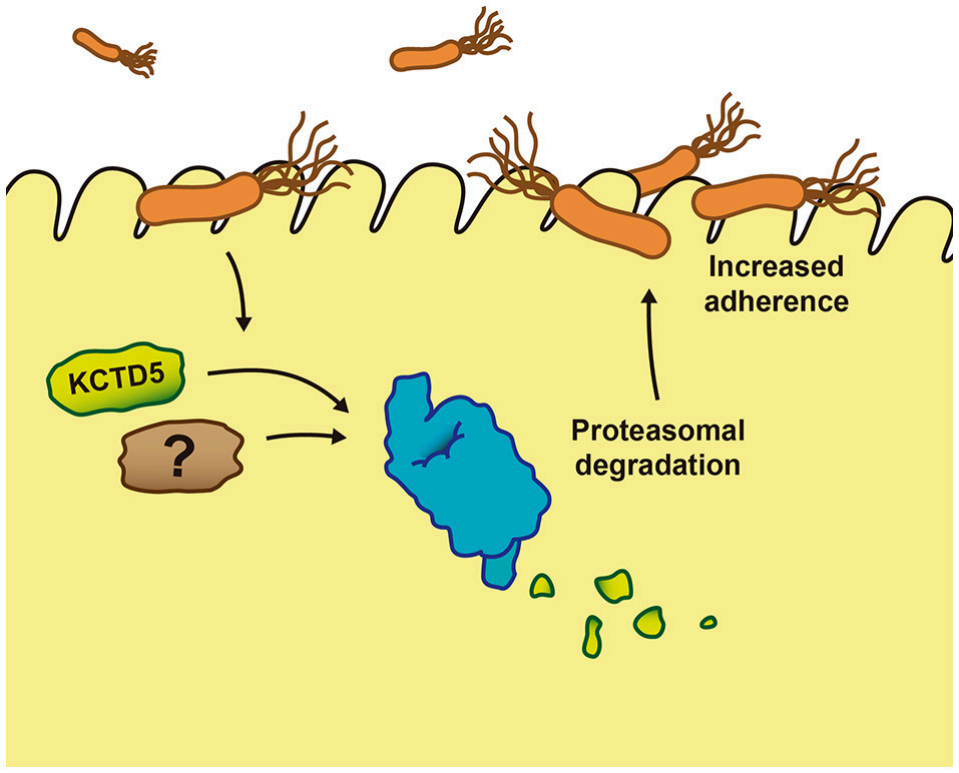
Proposed mechanism of the effect of KCTD5 levels during *Helicobacter pylori* infection. The *H. pylori* attach to the gastric epithelium cells induces the proteasome proteolysis of KCTD5 and/or other proteins facilitating the adherence of the bacterium.

Together, our data suggest that the *H. pylori* infection manipulates the UPS pathway and increases KCTD5 degradation, through a proteasome-dependent and ubiquitin-independent pathway. These mechanisms might facilitate the adhesion of this microorganism to establish a stable colonization in the gastric epithelium, contributing to the increased risk of gastric cancer associated with chronic *H. pylori* infection (Figure 8). Thus, these data provide novel insight regarding to the host machinery involved in *H. pylori* adherence infection. Moreover, these results might be used for design and develop of novel therapeutic strategies, which support the use of antibiotics to inhibit and diminish the viability of the bacteria in the host organism during colonization processes.

## Author contributions

Study idea and design: OC and HT; experimental work: AA, FU, JC, CR, MP, AS, MA, and OA. Paper concept and writing: AA, JC, OC, and HT. All authors discussed the results and commented the manuscript in all stages.

### Conflict of interest statement

The authors declare that the research was conducted in the absence of any commercial or financial relationships that could be construed as a potential conflict of interest.
