# Supplementary material for: KCTD5 and Ubiquitin Proteasome Signaling Are Required for Helicobacter pylori Adherence
Source: Front Cell Infect Microbiol. 2017 Oct 24;7:450. doi: 10.3389/fcimb.2017.00450 (PMC5660694; doi:10.3389/fcimb.2017.00450)
Supplement: Supplementary file 1 [file DataSheet1.DOCX]

Supplementary Material

KCTD5 and Ubiquitin Proteasome Signaling are required for *Helicobacter pylori* adherence

**Alhejandra Álvarez, Felipe Uribe, Jimena Canales, Cristóbal Romero, Andrea Soza, María Alicia Peña, Marcelo Antonelli, Oscar Almarza, Oscar Cerda* and Héctor Toledo*.**

*** Correspondence:**Oscar Cerda
oscarcerda@uchile.cl

Héctor Toledo

htoledo@med.uchile.cl

# Supplementary Figures


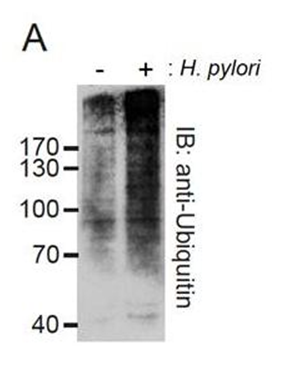


**Figure S1.** ***H. pylori* infection induces ubiquitination of different protein targets.** Immunoblot of lysates from AGS cells infected with *H. pylori*. Cells were transfected with HA-Ubi plasmid. (Multiplicity of infection, MOI=100) for 8 h.


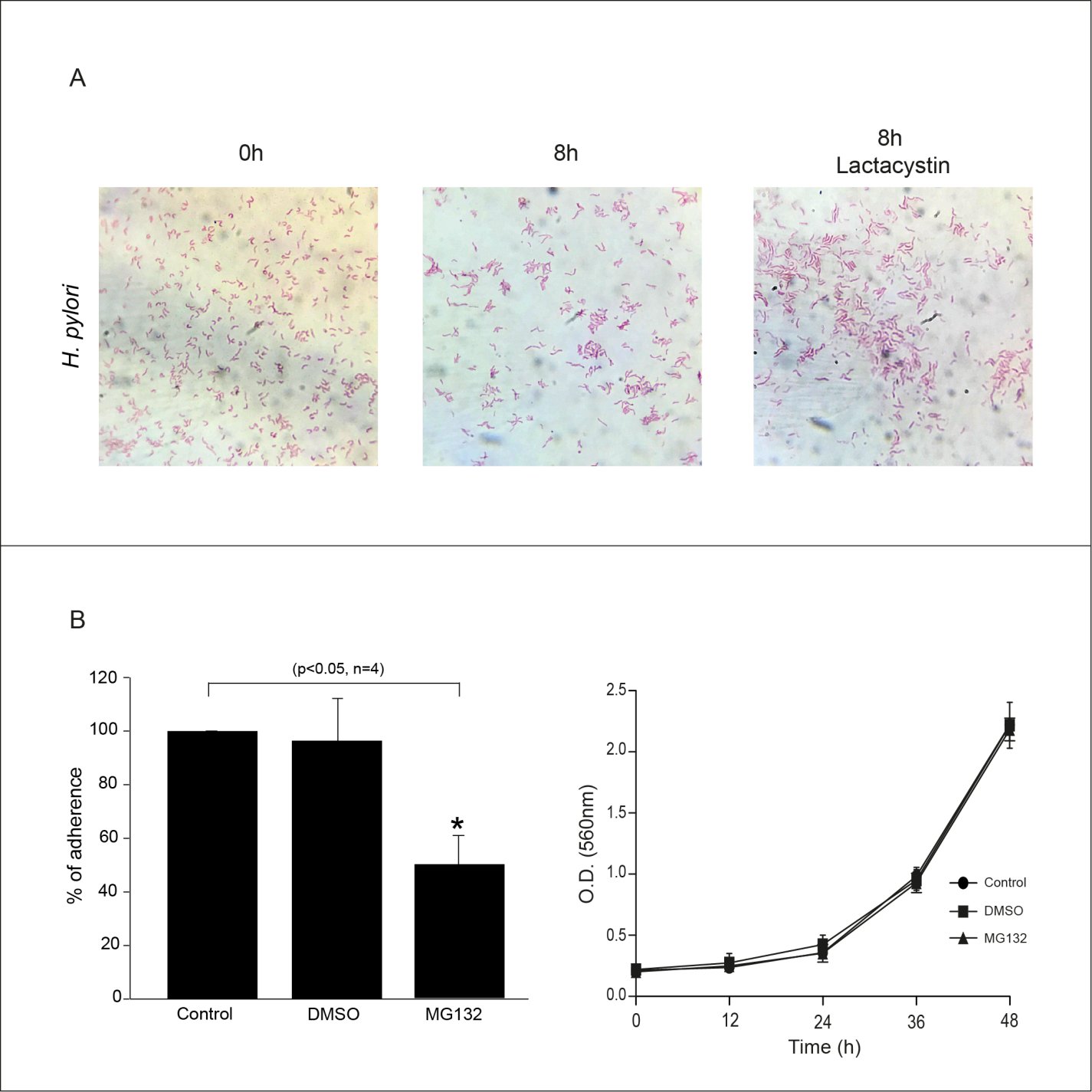


**Figure S2. Proteasomal inhibition reduces the *H. pylori* adhesion, but not bacterial survival. A.** Gram staining of *H. pylori* during the treatment of Lactacystin for 8 hours. **B.** Quantification of the *H. pylori* adhered to the AGS cell in presence of DMSO or 100 nM MG-132 (left). Growth curve of *H. pylori* in presence of DMSO and 100 nM MG-132. Untreated bacteria were included as controls (right).*: significant difference (p<0.05) versus untreated control. Statistical analysis was performed using a one-way ANOVA test.
